# Continuous versus interrupted anastomotic technique for the hepaticojejunostomy: a prospective cohort study

**DOI:** 10.1097/MS9.0000000000001902

**Published:** 2024-03-05

**Authors:** Tek Narayan Yadav, Narendra Pandit, Kunal Bikram Deo, Lalijan Awale, Durga Neupane, Shailesh Adhikary

**Affiliations:** a Department of Surgical, Gastroenterology B. P. Koirala Institute of Health Sciences, Dharan; b Department of Surgical, Gastroenterology Birat Medical College Teaching Hospital (BMCTH), Budhiganga, Morang, Nepal

**Keywords:** Anastomosis, bile leak, continuous suture, Hepaticojejunostomy, surgery, suture time

## Abstract

**Background:**

Interrupted sutures is the gold standard technique of hepaticojejunostomy (HJ) for bilioenteric anastomosis. This study compares the safety and early complications of continuous and interrupted suture HJ.

**Methods:**

A prospective study involving all elective HJ between September 2019 and June 2021 was conducted. Patients with type IV or V biliary strictures, duct diameter less than 8 mm and/or associated vascular injury, and bilateral HJ were excluded. The study patients were divided into two random groups; interrupted and continuous anastomotic technique. Patient demographics, preoperative parameters including pathology (benign vs. malignant), HJ leak, suture time, and postoperative morbidity were recorded.

**Results:**

Total 34 patients were enroled. Eighteen (52.9%) were into interrupted and 16 (47.1%) patients into the continuous group. Both the groups were comparable with regards to demographics, haemoglobin, serum albumin, preoperative cholangitis and biliary stenting. Total three (8.8%) patients in the entire study developed bile leak; interrupted-2 and continuous-1, which was not significant statistically (*P*=1.0). Similarly, total number of sutures used and the mean operating time to complete anastomosis in the continuous group was significantly lesser than the interrupted group (2.3±0.5 versus 9.6±1.6, *P*<0.001) and (16.2±3.1 versus 38.6±9.2 min, *P*<0.001), respectively. There were three (18.8%) re-exploration in the continuous anastomotic technique. Among them, only one re-operation was due to HJ anastomosis failure without mortality, remaining had re-exploration for bleeding (non-HJ).

**Conclusions:**

Both the techniques is safe with comparable morbidity. Further, continuous has an added advantage of decreased anastomotic time and cost.

## Introduction

Highlights

- Interrupted sutures is the gold standard technique of hepaticojejunostomy (HJ) for bilioenteric anastomosis.
- This study compares the safety and early complications of continuous and interrupted suture HJ.
- A prospective study involving all elective HJ between September 2019 and June 2021 was conducted.
- Both the techniques are safe with comparable morbidity. Further, continuous has an added advantage of decreased anastomotic time and cost.

Hepaticojejunostomy (HJ) is an important step in various hepatopancreatobiliary (HPB) surgeries such as pancreatoduodenectomy, bile duct resection, major liver resection, and liver transplantation^1,2^.

The most important complications following HJ are bile leak and anastomotic stricture^3,4^. Various literature has shown the leak rates between 2.3 and 5.6%^5–7^. Similarly, anastomotic stricture (late complication) develops in 3.7–8.0%^8^. The HJ can be performed using the interrupted suture (IS) or continuous suture (CS) technique. The time tested and the gold standard had been interrupted HJ as described by Bismuth *et al.* ^1^ Recently, continuous HJ has also been described with comparable results. Both have their advantages and disadvantages. IS technique has its universal use even in small diameter bile ducts. Despite this, the estimated cost and operating time of anastomosis seems to be higher than the CS technique. Large diameter bile ducts offer better sealing of the anastomosis when the CS technique is employed^9,10^.

Despite HJ’s frequent necessity in a varied spectrum of surgeries and relevant consequences of bile leak, anastomotic stricture and its sequel, only a few prospective studies compare IS and CS technique^9,10^. Therefore, we conducted a prospective comparative study where HJ outcomes between IS and CS techniques were compared.

## Material and methods

The study is a prospective comparative study conducted in the Division of Surgical Gastroenterology between September 2019 and June 2021. The study was approved by the Institute Review Committee-BPKIHS (IRC- 1527/019) and all participants in this study signed a written informed consent form, and the confidentiality of participant information was maintained. All patients scheduled for elective HJ and fulfilled inclusion/ exclusion criteria were included in the study. The inclusion criteria were patients who underwent HJ for benign or malignant HPB diseases. Those patients with high benign biliary stricture [Strasberg’s Type 4 or above], duct diameter less than 8 mm, bile duct injury associated with vascular injury, and bilateral HJ were excluded. They were excluded because of the inherited high-risk group for the anastomotic leak. The patients were divided into two groups by non-randomization technique; Interrupted HJ (Group 1) and Continuous HJ (Group 2).

A detailed history and examination findings, ECOG (Eastern Co-operative Oncology Group) performance status, BMI, and co-morbidities were noted. Blood investigations, including haemoglobin, serum albumin, and liver function tests were reviewed and noted. Indication/pathology for which HJ was done were entered. Those patients who were in cholangitis or required preoperative biliary drainage were noted. For allocation of a sample, operating surgeon’s preference was used at the time of surgery to divide into the two groups—interrupted (Group 1) and continuous anastomotic (Group 2).

### Anastomotic technique

An experienced chief HPB surgeon performed the HJ. Both the biliary and jejunal side stoma of appropriate size was prepared before starting the anastomosis. The anastomosis was performed using a fine monofilament polydioxanone 4-0 suture, a delayed absorbable suture, based on surgeon preference.

#### Interrupted anastomosis

First anterior layer suture on the bile duct side was pre-placed and rail-loaded. Then the posterior layer suture taking full-thickness bite through jejunal wall and bile duct was pre-placed and rail-loaded. After posterior layer completion, the sutures were tied sequentially with the knots inside the anastomosis. After that, anterior layer sutures pre-placed on the biliary side were used to take bites on the jejunal stoma’s anterior wall and sequentially tying the knots outside the wall to complete the anterior layer. At completion, a white gauze was placed at the anastomotic site to check for bile leak, and when present, tackled with re-enforced suture.

#### Continuous anastomosis

Anastomosis started from the left bile duct and bowel corner. The corner knot was tied outside, needle brought inside the bowel lumen, and continuous posterior wall anastomosis performed by sequential bowel wall bite and bile duct bites at a distance of 2 mm. On reaching the right corner, it was held taut with mosquito forceps. Similarly, anterior layer anastomosis was initiated from the left corner and sequentially bought to the right edge of the bile duct and bowel wall. Finally, the posterior wall thread and anterior wall thread was tied. Bile leak was checked using a white gauge after the anastomosis.

The stoma size was preferably 1.5 cm size, tension-free, using meticulous technique. The peri-anastomotic site was drained to look for a postoperative bile leak.

Bile leak was the primary outcome of the study. It was defined as fluid with an increased bilirubin level (at least three times greater than the serum bilirubin) in the abdominal drain or the intra-abdominal fluid on or after postoperative day 3, or the need for radiologic intervention for biliary collections or re-laparotomy resulting from bile peritonitis. The bile leak were graded according to criteria given by the International Study Group on Liver Surgery (ISGLS)^11^. The bile leak was divided into three grades as per the severity. Grade A was bile leakage requiring no or little change in the patient’s management. In contrast, Grade B required interventional procedures such as percutaneous catheter drainage (PCD) but manageable without re-laparotomy or bile leak lasting for more than one week, and Grade C as bile leak which required re-exploration. Any event of bile leak in the postoperative period and its grading were recorded. Any interventions required in the form of percutaneous catheter drainage or re-exploration was entered.

The secondary outcome of the study was time duration to complete HJ anastomosis (in minutes) and the total number of sutures required for the anastomosis. All complications were noted and graded as per the Clavien-Dindo classification^12^. The Clavien-Dindo grade I and II were classified as minor morbidity; whereas grade greater than or equal to III were classified as major morbidity. Morbidity related to bile leak was separately noted. The 30-and 90-day mortality related to bile leak and the non-bile leak were noted.

The patients were followed-up for at least six months post-surgery at an out-patient department by history/examination, liver function test, and abdominal ultrasound for any delayed complications; early anastomotic stricture. The clinically significant anastomotic stricture was defined by the presence of pain, cholangitis, jaundice, abnormal liver function tests, and focal narrowing at the HJ site as seen on imaging.

### Statistical analysis

Collected data were entered in the Microsoft excel 2007, and was converted into SPSS 11.5 version for statistical analysis. For descriptive statistics, percentage, mean, standard deviation, median, interquartile range, minimum and maximum values were calculated. For interferential statistics, the chi-square test or Fischer’s exact test (when one or more cells had a count less than 5) were used for the categorical outcome. Independent t-test (for normally distributed data) or Mann–Whitney U test (for non-normal distribution) was applied to compare the numerical outcome between groups. These tests were used to determine the significant differences between groups and other selected sociodemographic, clinical parameter, laboratory parameter, and treatment outcomes at 95% CI, where the level of significance was considered *P* less than 0.05.

The work has been reported in line with the STROCSS guidelines^13^.

## Results

**Figure 1:**
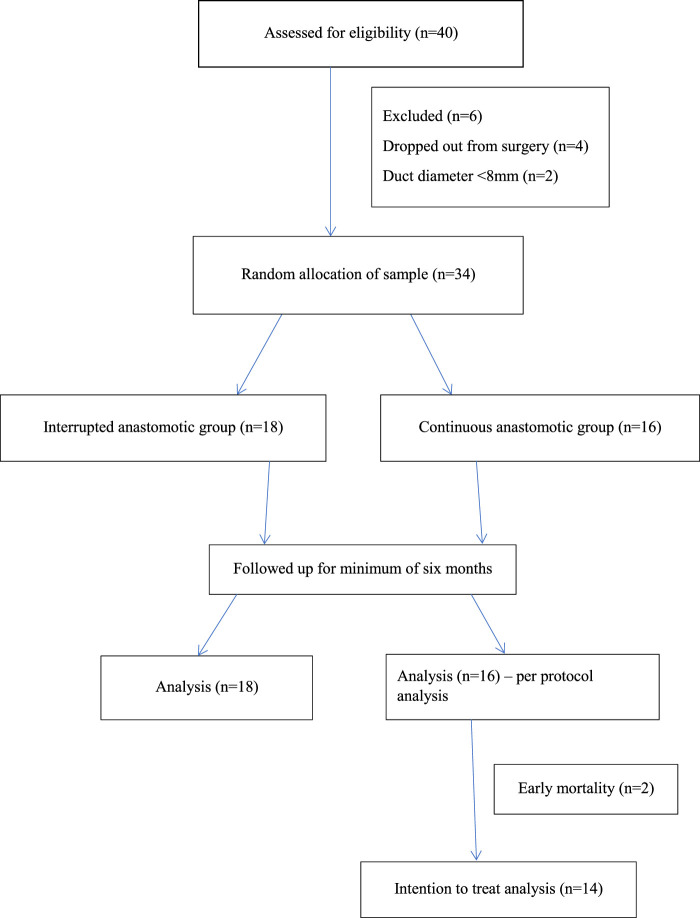
Consort diagram of the study.

Total 40 patients were assessed for eligibility, six were excluded and the remaining 34 were allocated into two groups. Eighteen (52.9%) were allocated into interrupted anastomotic group and 16 (47.1%) patients into continuous anastomotic group as shown in Fig. 1.

### Patient characteristics

The mean age of the patient in the entire study was 50.5±15.5 years (range: 18–75) with male preponderance (M: F=3.2:1). Patient’s demographic parameters concerning age, sex, BMI, and co-morbidities between the two groups were comparable (*P*>0.05). Preoperative haemoglobin (Hb) and albumin level between both the groups were also comparable (Hb 11.2±1.2 versus 10.1±1.0 g/dl, *P*=0.63 and albumin 3.9±0.5 versus 3.5±0.3 g/dl, *P*=0.33). There was no statistical difference between the groups when preoperative cholangitis (22.2% versus 18.8%, *P*=1.0) and preoperative stenting (16.7% versus 6.2%, *P*=0.6) were analyzed.

There was an equal number of benign and malignant disease pathology in the interrupted group [9 (50%) versus 9 (50%)]. In the continuous group, there was a higher number of malignant disease compared to benign [13 (81.2%) versus 3 (18.8%)]. This was because of two patients being cross-over by the intraoperative decision for continuous anastomosis. However, this was not statistically significant (*P*=0.06) (Table 1).

**Table 1:** Patient characteristics between two groups.

	Interrupted group (*n*=18)	Continuous group (*n*=16)	*P* value
Age, years (mean±SD)	48.0±17.8	53.2±12.3	0.33
Sex, *n* (%)			
Male	13 (72.2)	13 (81.2)	0.69
Female	5 (27.8)	3 (18.8)	
Co-morbidities, *n* (%)			
Diabetes	4 (22.2)	3 (18.8)	1.0
Hypertension	5 (27.8)	2 (12.5)	0.40
Chronic kidney diseases	—	1 (6.2)	0.47
BMI, kg/m^2^ (mean±SD)	20.7±2.1	19.5±2.6	0.14
ECOG, *n* (%)			0.06
0	5 (27.8)	0	
1	5 (27.8)	3 (18.8)	
2	8 (44.4)	12 (75.0)	
3	0	1 (6.2)	
Preoperative cholangitis, *n* (%)	4 (22.2)	3 (18.8)	1.0
Preoperative stenting, *n* (%)	3 (16.7)	1 (6.2)	0.60
Haemoglobin (Hb), gm/dl (mean±SD)	11.2±1.2	10.1±1.0	0.63
Albumin, gm/dl (mean±SD)	3.9±0.5	3.5±0.3	0.33
Diagnosis, *n* (%)			
Benign	9 (50.0)	3 (18.8)	0.06
Hepatolithiasis (3)			
Choledochal cyst (5)			
Chronic pancreatitis (1)			
Post-cholecystectomy biliary stricture (3)			
Malignant			
Pancreatic head cancer (6)	9 (50.0)	13 (81.2)	
Periampullary carcinoma (8)			
Hilar cholangiocarcinoma (2)			
Gallbladder cancer (6)			

ECOG, Eastern Co-operative Oncology Group.

### Primary outcome

Total three (8.8%) patients in the entire study group developed bile leak; interrupted-2 and continuous group-1. Two (11.1%) patients from the Group 1 had a minor leak on a postoperative day two and day three, respectively, as revealed from the drain site. Daily drain output was between 30 and 40 ml and remained for three days. The leak was grade A which subsided on its own on POD 5 and 6, respectively, and did not require any further intervention. One (6.2%) patient from the Group 2 developed a major (grade C) bile leak. It was revealed on day two as bile leak from the drain site with associated peritonitis and systemic disturbances (tachycardia, tachypnea). The patient underwent re-operation on the second postoperative day. The operation includes tube hepaticostomy from the defunctionalized distal jejunal limb, suture closure of the anastomosis dehiscence (1 cm size), peritoneal lavage, and drainage of the subhepatic region. The patient improved with it. There was no statistical difference concerning bile leak between the two groups (*P*=1.0) (Table 2). Similarly, two patients from the Group 2 had early postoperative mortality (Day 2 and day 4) and thereby could not be assessed for bile leak. When excluded in intention to treat analysis (*n*=14), the bile leak rate was 7.1% (Table 3).

**Table 2:** Comparison of primary outcome between two groups (per-protocol analysis).

	Interrupted group (*n*=18)	Continuous group (*n*=16)	*P* value
Bile leak, *n* (%)	2 (11.1)	1 (6.2)	1.0
Grade A	2 (11.1)	—	
Grade B	—	—	
Grade C	—	1 (6.2)	

**Table 3:** Comparison of primary outcome between two groups (intention to treat analysis).

	Interrupted group (*n*=18)	Continuous group (*n*=14)	*P* value
Bile leak, *n* (%)	2 (11.1)	1 (7.1)	0.87
Grade A	2 (11.1)	—	
Grade B	—	—	
Grade C	—	1 (7.1)	

### Secondary outcomes

The total number of sutures utilized to complete the anastomosis in the continuous technique group was significantly lesser than the interrupted group (2.3±0.5 versus 9.6±1.6, *P*<0.001). This significantly reduced the estimated cost incurred due to suture when utilizing continuous anastomotic technique (Nepalese Rupees 2,375.0±500.0 versus 9,611.1±1,685.1, *P*<0.001). Furthermore, the mean operating time taken to complete the HJ anastomosis using continuous technique was significantly lower than the interrupted technique (16.2±3.1 versus 38.6±9.2 min, *P*<0.001) (Table 4).

**Table 4:** Secondary outcomes compared between interrupted and continuous anastomotic technique.

	Interrupted group (*n*=18)	Continuous group (*n*=16)	*P* value
No. sutures used (mean±SD)	9.6±1.6	2.3±0.5	<0.001
Duration of anastomosis, in minutes (mean±SD)	38.6±9.2	16.2±3.1	<0.001
Estimated blood loss, in ml (mean±SD)	256.1±226.0	319.3±219.5	0.41

The re-exploration was required in three (18.8%) patients in the continuous anastomotic technique group as compared to none in the interrupted group (*P*=0.09). Among them, only one re-operation was due to HJ anastomosis failure leading to major bile leak and biliary peritonitis. Two of the patients had re-exploration for bleeding. None of the bleedings was from the HJ site.

## Discussion

In the present study, where we compared interrupted with a continuous anastomotic technique for HJ, the rate of bile leak was similar in both the groups [interrupted vs. continuous 2 (11.1%) versus 1 (6.2%), *P*=1.0]. There was no mortality related to bile leaks in any of the groups. Morbidity related to bile leak was comparable in both the groups (*P*=1.0). However, the number of sutures utilized and the time duration required to perform continuous HJ were significantly less compared to the interrupted anastomotic technique (*P*<0.001).

The most common indication for performing HJ in modern era is malignant disease. Carcinoma head of the pancreas and periampullary carcinoma are the commonest pathology with the dilated and thick walled ducts^14,15^. There seems to be some form of bias in this study, as more malignant disease cases were undergoing continuous anastomosis. A probable reason for this may be dilated and thick walled duct in malignant pathology compared to non-thick-walled duct in benign pathology as observed by the operating surgeon intraoperatively. Surgeons are more comfortable doing continuous anastomosis in the dilated and thickened duct, and there have been cross-over or intraoperative change in the decision to perform CS (two patients) versus IS technique.

The most important short term complication following HJ is bile leak. It can lead to biliary peritonitis, biloma, abscess, sepsis, and wound-related complications^16,17^. In our study, a total three (8.8%) patients developed bile leak. Two (11.1%) patients from the interrupted anastomotic group had a minor leak (Grade A), which subsided on its own in three days and did not require any additional intervention. One (6.2%) patient from continuous anastomotic group developed a major (grade C) bile leak requiring re-operation. There was no statistical difference concerning bile leak between the two groups (*P*=1.0). This is the major advantage of interrupting anastomosis, as major dehiscence of the anastomosis is probably unlikely due to the retained strength of anastomosis by interrupted technique. Contrarily, the dehiscence is large and major in continuous, requiring re-exploration as seen in the present case.

Bile leak (8.8%) in our study was in line with other studies conducted by Tatsuguchi and Seifert L and colleagues, which had an overall bile leak rate of 6–14%^10,18^. There was no difference in leak rate between interrupted and continuous anastomotic arm as seen in their study as well. Another retrospective study by Mohamed *et al*.^15^ found bile leak in the continuous anastomotic arm to be 5% and in the interrupted anastomotic arm to be 8.6%. A recent study by Guangbing and colleagues has shown less leak rate with continuous technique than interrupted technique (1.67% versus 11.67%)^2^. The slightly higher rate of anastomotic leak in our study was probably due to the small sample size in the study. Further, the most important thing to prevent bile leaks is not the anastomotic technique that is employed, rather the meticulous technique along with the use of fine sutures by experienced HPB surgeon at the high-volume academic centre as is ours.

In our study, the total number of sutures used to complete anastomosis in the continuous technique group was significantly lesser than the interrupted group (2.3±0.5 versus 9.5±1.6, *P*<0.001). Similarly, there was a reduced estimated cost incurred and the operating time due to the suture when utilizing continuous anastomotic technique. The reduced anastomotic time would technically reduce the overall operative time in similar surgical cases and thereby avoid the consequences of a prolonged operative period. Similar results were demonstrated by the other authors on operating time, costs of sutures which is further beneficial to the patients^9,10^.

None of the patients from either group developed anastomotic stricture during the follow-up. Various studies have shown the median time to development of anastomotic stricture to be 8–12 months^19,20^. However, due to the short duration of the study and follow-up, we could not follow the patients over a longer period to assess for the development of late anastomotic stricture. The rate of anastomotic stricture in different studies has been shown as 3–12%^21,22^. Further extended duration of follow-up is required to find out the rate of delayed anastomotic stricture.

The study is limited by the non-randomization of the patients, small sample size, heterogenous group, and lack of long-term follow-up to observe for the delayed stricture. Because of small sample size in both groups, the study did not compare the patient factors (co-morbidity, haemoglobin, serum albumin, jaundice) affecting the bile leak between the two groups. Further, there was non-uniform distribution of benign and malignant cases in the continuous anastomotic group, leading to biasness. However, it is the first prospective study conducted in our part to compare the outcomes and its advantages. Further, to remove all these biases, proper randomization, large sample size, homogenous patient groups from multi-centre is required to better study the primary outcome.

## Conclusion

This is one of the fewer prospective studies which compared interrupted and continuous anastomotic technique for performing HJ. The study has shown that there is no difference in the rate of bile leak between the two groups, and morbidity and mortality related to bile leak were similar, with the added advantage of lesser anastomotic time and suture requirement with continuous anastomotic technique. Further randomized prospective studies with large sample size and long-term follow-up can be taken up in the future.

## Ethical approval

Ethical approval was obtained from B. P. Koirala Institute of Health Sciences (BPKIHS), ethical clearance committee with an IRB assigned number of 1527/019.

## Consent for publication

Not applicable.

## Sources of funding

Non received.

## Author contribution

T.N.Y.: contributed to the source, data collection, design, analysis, interpretation of the research manuscript. N.P. contributed to the inception, design, analysis, interpretation, and drafting of the research manuscript. L.A.: contributed to the design, and analysis of the research manuscript. K.B.D., D.N. and S.A. contributed to the analysis, interpretation, and drafting of the research manuscript. All authors read and approved the manuscript for publication. T.N.Y. is the first author and N.P. is the corresponding author.

## Conflicts of interest disclosure

The authors declare that there are no financial and non-financial competing interests.

## Research registration unique identifying number (UIN)

Research registration is not done.

## Guarantor

Narendra Pandit.

## Availability of data and materials

The datasets used and/or analyzed during the current study are available from the corresponding author upon reasonable request.

## Provenance and peer review

Not commissioned, externally peer-reviewed
